# Blood-CNS barrier dysfunction in amyotrophic lateral sclerosis: Proposed mechanisms and clinical implications

**DOI:** 10.1177/0271678X231153281

**Published:** 2023-01-26

**Authors:** Moritz Steinruecke, Rebecca Murphy Lonergan, Bhuvaneish T Selvaraj, Siddharthan Chandran, Blanca Diaz-Castro, Maria Stavrou

**Affiliations:** 1 Edinburgh Medical School, The University of Edinburgh, Edinburgh, UK; 2 University of Cambridge School of Clinical Medicine, Cambridge, UK; 3 North Middlesex University Hospital NHS Trust, London, UK; 4 Euan MacDonald Centre for MND Research, The University of Edinburgh, Edinburgh, UK; 5 Centre for Clinical Brain Sciences, The University of Edinburgh, Edinburgh, UK; 6 Dementia Research Institute at The University of Edinburgh, Edinburgh, UK

**Keywords:** Amyotrophic lateral sclerosis, astrocytes, blood-CNS barrier, mitochondria, neuroinflammation

## Abstract

There is strong evidence for blood-brain and blood-spinal cord barrier dysfunction at the early stages of many neurodegenerative diseases, including amyotrophic lateral sclerosis (ALS). Since impairment of the blood-central nervous system barrier (BCNSB) occurs during the pre-symptomatic stages of ALS, the mechanisms underlying this pathology are likely also involved in the ALS disease process. In this review, we explore how drivers of ALS disease, particularly mitochondrial dysfunction, astrocyte pathology and neuroinflammation, may contribute to BCNSB impairment. Mitochondria are highly abundant in BCNSB tissue and mitochondrial dysfunction in ALS contributes to motor neuron death. Likewise, astrocytes adopt key physical, transport and metabolic functions at the barrier, many of which are impaired in ALS. Astrocytes also show raised expression of inflammatory markers in ALS and ablating ALS-causing transgenes in astrocytes slows disease progression. In addition, key drivers of neuroinflammation, including TAR DNA-binding protein 43 (TDP-43) pathology, matrix metalloproteinase activation and systemic inflammation, affect BCNSB integrity in ALS. Finally, we discuss the translational implications of BCNSB dysfunction in ALS, including the development of biomarkers for disease onset and progression, approaches aimed at restoring BCNSB integrity and *in vitro* modelling of the neurogliovascular system.

## Main text

There is strong evidence for blood-brain and blood-spinal cord barrier (BBB, BSCB) dysfunction at the early stages of many neurodegenerative diseases (NDDs), including amyotrophic lateral sclerosis (ALS).^1,2^ Since impairment of the blood-central nervous system barrier (BCNSB) occurs during the pre-symptomatic stages of ALS, the mechanisms underlying this pathology are likely also involved in the ALS disease process. In this review, we explore how drivers of ALS disease, particularly mitochondrial dysfunction, astrocyte pathology and neuroinflammation, may contribute to BCNSB impairment. Additionally, while the BCNSB is often considered to be an obstacle to clinical intervention, its disruption in ALS presents diagnostic and therapeutic opportunities.

## The blood-central nervous system barrier

The BCNSB separates the brain and spinal cord parenchyma from the general circulation. This barrier controls the influx and efflux of substances to maintain the CNS microenvironment, which is required for nervous system function.^3^ The BCNSB facilitates the transport of amino acids, vitamins and sugars via carrier-mediated mechanisms while larger molecules such as lipoproteins and hormones cross using specific receptors. The barrier also restricts the movement of neurotoxins and blood cells into the CNS and clears toxins via efflux pumps.^1^

These unique properties arise from the cellular and molecular components of the BCNSB which control the para- and transcellular movement of substances.^4^ Endothelial cells (ECs) of cerebral capillaries are connected by tight and adherens junctions (TJs, AJs) which limit the paracellular movement of toxic substances.^5^ The key TJ proteins are occludin, claudins and zona occludens-1 (ZO-1). ECs secrete the glycocalyx, also known as the pericellular matrix, which covers the luminal surface of blood vessels and is made up of glycosaminoglycans. A basement membrane surrounds endothelial cells and is in contact with pericytes, astrocytes and microglia.

Pericytes, which adhere to ECs, are key contributors to BCNSB integrity and regulate transcellular vesicular transport, matrix metalloproteinase (MMP) secretion and blood vessel dilation.^6^ Pericytes and ECs are embedded in an extracellular matrix rich in collagen, fibronectin, and laminin proteins, which is a large source of growth factors.^7^ Astrocyte endfeet are terminal processes which surround virtually all capillary surfaces to form the glia limitans and, among other roles, regulate water, glucose, and electrolyte homeostasis. Perivascular microglia, the resident immune cells of the brain, monitor the parenchyma for blood-derived toxins and inflammatory stimuli that have penetrated the BCNSB. Neurons rarely make direct contact with the BCNSB but receive and deliver signals via pericytes and astrocytes, which have the capacity to modulate synapses.^1^

While there is significant structural and functional overlap between the BSCB and BBB, there are a few key differences. ECs at the BSCB express fewer TJ proteins than those at the BBB, making the BSCB more permeable, particularly to pro-inflammatory cytokines.^8^ This leaves the BSCB more vulnerable to infiltration and susceptible to disease. The efflux protein p-glycoprotein is also downregulated at the BSCB, causing toxins that cross the barrier to be removed more slowly.

The neurogliovascular unit (NGVU) is a functional structure composed of vascular cells, glial cells, neurons and an extracellular matrix which controls the blood supply to CNS tissue.^9^ Several components of the NGVU form part of the BCNSB and therefore provide useful insight into cellular and molecular interactions at the barrier. Similar to the BCNSB, the NGVU has been found to be dysfunctional in many NDDs and it is likely that these events share pathophysiological mechanisms.

**Figure 1.**
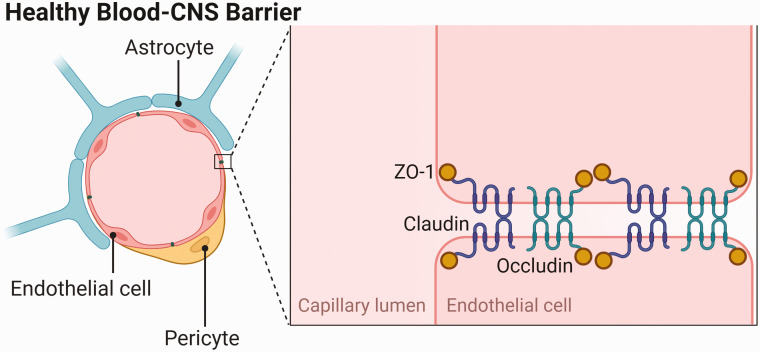
Components of the healthy blood-central nervous system barrier, including the tight junction proteins zonula occludens-1 (ZO-1), claudin and occludin. Adapted from “Endothelial Junctions in the Blood Brain Barrier”, by BioRender.com (2022). Retrieved from https://app.biorender.com/biorender-templates.

There are several ways of evaluating BCNSB function in disease. Although many studies focus on BCNSB hyperpermeability, there are other important features of the barrier which are likely dysregulated in NDDs. These include the transport of nutrients required for neuronal function and mechanisms by which the BCNSB clears toxins and waste products from the nervous system (Figure 1).

## Amyotrophic lateral sclerosis

ALS, the most common motor neuron disease, is a multisystem NDD characterised by progressive degeneration of upper and lower motor neurons. ALS has an earlier onset than other NDDs, between the ages of 51 and 66 years, and carries a worse prognosis, with a median survival time of only 2–4 years from diagnosis.^10^ Patients generally present with either muscular weakness of the limbs, or bulbar symptoms, such as dysarthria and dysphagia. Up to 50% of patients also develop behavioural and/or cognitive impairment over the disease course. Most patients with ALS die from respiratory insufficiency.

The majority of ALS cases are sporadic (sALS), with approximately 10% caused by monogenic mutations (familial ALS, fALS). The most common of these mutations are in the *C9orf72*, *SOD1*, *TARDBP* (*TDP*) and *FUS* genes, which have multisystem effects.^11^ These risk genes are preferentially expressed by motor neurons, which are targeted in ALS, but they are also expressed by non-neuronal cells at the BCNSB, including astrocytes and microglia.^12,13^ As sALS and fALS are phenotypically and pathologically indistinguishable, generalisable conclusions can be drawn from studies of fALS. The most common genetic causes of ALS are a hexanucleotide repeat expansion in the *C9orf72* gene and missense mutations in the *SOD1* gene. C9orf72 is part of the differentially expressed in normal and neoplastic cells (DENN) family of proteins and is thought to regulate endosomal trafficking and autophagy in neurons.^14,15^ Since being discovered, *C9orf72* repeat expansions have implicated aberrant RNA processing, metabolic pathways and proteostasis in the ALS disease process.^16^ Likewise, mutations in the *SOD1* gene reduce the enzymatic antioxidant function of SOD1, highlighting the role of oxidative stress and mitochondrial dysfunction in ALS.^17^*TDP-43* mutations also disrupt RNA regulatory mechanisms, including transcriptional regulation, alternative splicing, and mRNA stabilisation.^18^

Genetic models, including both transgenic and physiological rodents, have proven influential in our understanding of the aetiopathogenesis of ALS.^19^ Transgenic approaches allow protein deposits to be studied by overexpressing proteins which are implicated in the disease process. Conversely, models which use gene targeting to express mutant genes at physiological levels help investigate disease onset and early-stage mechanisms. SOD1-G93A was the first transgenic mouse line to be used for the study of ALS and displays rapidly progressive motor neuron loss and limb paralysis.^20^ In particular, several non-cell autonomous and neuroinflammatory mechanisms have been replicated using the SOD1 mouse model.^21^ However, other features of human ALS, such as occasional bulbar onset and degeneration, are not seen in SOD1 rodents.^22^ While these models do not completely mirror the disease phenotype seen in humans, they do allow for detailed mechanistic study of neurodegeneration and screening of drug targets.

It is now acknowledged that ALS patients experience a pre-symptomatic disease phase which is associated with cellular and molecular changes but without clinical manifestations.^23^ Understanding the pre-symptomatic stages of ALS is critical for the identification of biomarkers to facilitate early diagnosis and pre-clinical treatment. This is especially important given that many ALS patients experience delays to diagnosis, which are associated with slowed access to symptomatic treatments, more frequent hospital admissions and shortened survival.^24^ Notably, both clinical and experimental studies provide strong evidence of BCNSB dysfunction, particularly changes in barrier permeability, prior to or at the very early stages of ALS symptom onset.^25^ Experimental restoration of barrier integrity has been shown to delay motor neuron dysfunction and death in an ALS model and reduces neuropathology in a model of multiple sclerosis (MS), highlighting its therapeutic applications.^26,27^

## BCNSB dysfunction in ALS and experimental models

### Evidence of BCNSB dysfunction in ALS

Alterations in the cellular and structural components of the BCNSB are responsible for the barrier disruptions seen in various neurological and CNS disorders. These changes affect the microenvironment of TJs, including their expression and distribution; dysregulate transport systems and enzymatic function; and disrupt the basement membrane, causing immune cell infiltration into the brain parenchyma, disturbances in CNS homeostasis, and tissue damage.^28^

Two studies in the 1980s demonstrated elevated levels of IgG, albumin and complement protein C3a in the cerebrospinal fluid (CSF) of ALS patients, providing evidence of BCNSB hyperpermeability.^29,30^ These findings were supported by evidence of IgG and C3 deposits in the motor cortex and spinal cord on autopsy.^31^ Perivascular and intraparenchymal infiltration of lymphocytes and cytokines, as well as immune cell activation, was then identified in human ALS spinal cords.^32,33^ In SOD1 mice, Evans blue dye leaks from spinal cord capillaries at an early stage of disease.^34^ These abnormalities may be caused by downregulation of GLUT1 and CD146, proteins which are key for endothelial cell function.^35^

Since then, several different proteins and cell types have been implicated in the observed BCNSB dysfunction in ALS. In SOD1 mice, ECs are highly vacuolated, and mitochondria within these cells show abnormal cristae morphology and degenerate.^36^ In 2008, Zhong et al. identified reduced TJ protein expression levels (ZO-1, occludin and claudin-5) between BSCB endothelial cells in this same model.^37^ These changes were observed before the onset of motor neuron degeneration and neuroinflammation. In support of these findings, another group demonstrated reduced mRNA expression of ZO-1 and occludin in the spinal cords of ALS patients.^38^ Levels of circulating endothelial cells are also reduced in moderate and severe ALS patients, which may be explained by a lack of endothelial shedding and/or impaired endothelial renewal.^39^

Pericytes regulate capillary permeability and blood flow at the BCNSB and pericyte-deficient mice experience reduced brain microcirculation and BBB impairment, which causes proteins and neurotoxic macromolecules to accumulate.^40^ Together, these changes cause vascular-mediated neurodegeneration. Notably, loss of pericyte-derived pleiotrophin (PTN), a neurotrophic growth factor, mediates these degenerative changes, and intracerebroventricular delivery of PTN rescues neuronal loss in pericyte-deficient mice.^41^ In ALS patients, pericytes are severely degenerated in the medulla and spinal cord.^42^ Pericyte number and coverage are also reduced at spinal cord capillaries, resulting in perivascular erythrocyte extravasation and fibrin accumulation.^43^ Further, electron microscopy studies have identified significant lipofuscin deposits within endothelial cells and pericytes, which cause cell death.^44^

Astrocytes, which normally form the cellular interface between neurons and the BCNSB, are also structurally and functionally affected in ALS. In human ALS tissue samples, astrocyte end-feet are degenerated and are dissociated from the endothelium in the spinal cord.^45^ At late disease stages in the SOD1 rat model, astrocytic processes, which surround blood vessels and are located near motor neurons, show increased aquaporin 4 (AQP4) expression and decreased expression of the potassium channel Kir4.1.^46^ These abnormalities likely affect BCNSB integrity by reducing the ability of astrocytes to maintain water and potassium homeostasis. Similar to endothelial cells, astrocytes show significant mitochondrial pathology in SOD1 mice. The basement membrane, which is found between astrocyte end-feet and endothelial cells, is also disrupted in ALS, as evidenced by the loss of laminin and the presence of collagen IV in microglia.^34,45^

Together, these abnormalities result in major impairment of the brain and spinal cord microvasculature in ALS. Blood flow to the cervical and lumbar spinal cord is reduced by 30–45% in SOD1 mice prior to symptom onset.^37^ Total capillary length in the lumbar spinal cord is also reduced by 10-15% before motor neuron loss and inflammatory changes occur. In the spinal cord grey matter of sALS patients, there is evidence of increased microvascular density, which may reflect a compensatory response to vascular insufficiency resulting from dysfunctional capillaries.^44^ This rapid angiogenesis could, however, be associated with further BCNSB hyperpermeability, exacerbating immune and inflammatory cell extravasation.

**Figure 2.**
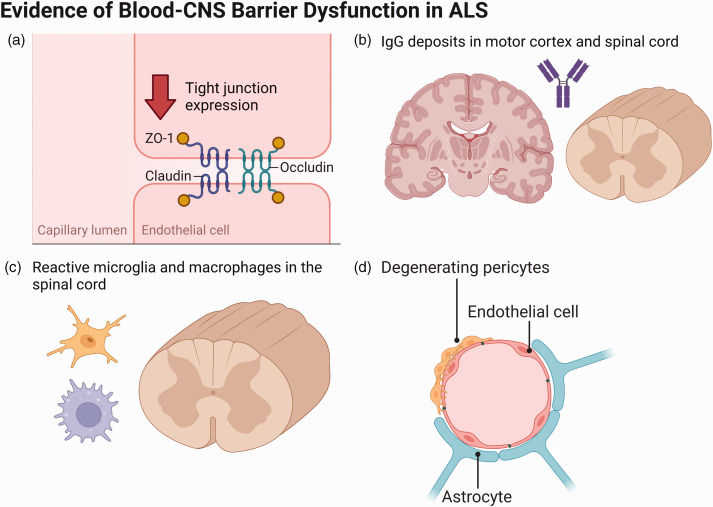
Evidence for blood-CNS barrier dysfunction in ALS, including (a) reduced tight junction protein expression, (b) IgG deposits in the motor cortex and spinal cord, (c) reactive microglia and macrophages in the spinal cord, and (d) pericyte degeneration in the medulla and spinal cord. Adapted from “Endothelial Junctions in the Blood Brain Barrier”, by BioRender.com (2022). Retrieved from https://app.biorender.com/biorender-templates.

Since BCNSB impairments arise early in the disease course, it is likely that the mechanisms initiating this dysfunction are closely related to the aetiopathogenesis of ALS. Of the mediators of BCNSB integrity discussed above, mitochondrial dysfunction, astrocyte pathology and neuroinflammation are known drivers of ALS disease. With this in mind, we next focus on how these three mechanisms contribute to both motor neuron pathology and BCNSB disruption (Figure 2).

### Mitochondrial dysfunction and oxidative stress

Mitochondrial dysfunction and oxidative stress are key mechanisms in the pathophysiology of ALS, specifically via defective mitochondrial respiration, reduced ATP production and excessive reactive oxygen species (ROS) accumulation.^47^ Mitochondria are also found at particularly high levels at the BCNSB and play a key role in maintaining barrier function, which suggests that mitochondrial pathology may contribute to the BCNSB abnormalities seen in ALS.^48^

Abnormal mitochondrial morphology is common in the motor neurons of patients with ALS and various SOD1 mouse models replicate this pathology.^49,50^ Many protein aggregates seen in ALS, such as FUS, interact directly with mitochondria, which leads to oxidative stress and motor neuron degeneration.^51^ Recently, mitochondrial bioenergetic deficits have been shown to cause motor neuron pathology in an induced pluripotent stem cell (iPSC) model of *C9orf72*-mediated ALS.^52^ Importantly, overexpression of peroxisome proliferator-activated receptor gamma coactivator 1α (PGC1α), a regulator of mitochondrial function and biogenesis, rescued the deficits in mitochondrial transport and axon length seen in this model. Some variants in the mitochondrial cristate gene *CHCHD10* are also associated with motor neuron degeneration and may cause ALS.^53^ As with BCNSB disruption, mitochondrial damage is demonstrated prior to disease onset in several experimental models.^49^ Further, ALS mouse models show disrupted mitochondrial cristae and degenerating mitochondria in ECs which form part of the BCNSB.^36^ Together, these lines of evidence strongly implicate structural and functional mitochondrial impairment in ALS aetiopathogenesis.

In health, mitochondria have important functions in maintaining BCNSB integrity. Mitochondria were first suggested to be involved in BCNSB function when they were found at high volumes in capillary endothelial cells from tissues associated with the BBB.^48^ This was linked to the high energetic demands of endothelial cells at the BBB due to their transport and barrier functions. The mechanistic role of mitochondria in maintaining barrier function has since largely been elucidated by studying the effects of mitochondrial pathology on BCNSB integrity.

In ischaemia-reperfusion injuries, aberrant mitochondrial function leads to BBB impairment. Doll et al. showed that a lipopolysaccharide (LPS) challenge in a model of ischaemic stroke decreases oxidative phosphorylation and expression of respiratory chain complexes in cerebrovascular endothelial cells.^54^ Pharmacological blockade of oxidative phosphorylation using mitochondrial inhibitors increased BBB permeability *in vitro* and *in vivo* and disrupted TJs in cultured cerebrovascular ECs. This worsens stroke outcomes in mice. The same group later demonstrated that overexpressing the microRNA miR-34a also increases BBB permeability by inhibiting mitochondrial function.^55^

Similarly, Haileselassie et al. used an LPS challenge to induce inflammation and BBB disruption in cell culture and mouse models of sepsis.^56^ Dynamin-related protein 1 (Drp1), which regulates mitochondrial fission and promotes mitochondrial fragmentation in stress conditions, showed increased activity and mitochondrial localisation following LPS exposure. Resulting mitochondrial defects were associated with BBB impairment. P110, an inhibitor of the interaction between Drp1 and Fission 1 (Fis1), attenuated this mitochondrial damage and reduced BBB hyperpermeability. Drp1 has also been shown to mediate BBB integrity following middle cerebral artery occlusion. Specifically, dexmedetomidine, a selective α_2_ adrenergic agonist, inhibits excessive mitochondrial fission in endothelial cells and ameliorates BBB disruption.^57^ Dexmedetomidine acts by indirectly phosphorylating serine 637 of Drp1. In two cell models of ALS, inhibition of the Drp1/Fis1 interaction using P110 has been shown to improve mitochondrial structure and function and reduce ROS levels. The same study showed that SOD1 mice treated with P110 from disease onset experienced improved motor performance and survival.^58^

In Alzheimer’s disease (AD), amyloid beta (Aβ) interacts directly with mitochondria. This causes defects in calcium handling and ROS production, leading to BBB and NGVU dysfunction.^59^ Excessive ROS production disrupts the BCNSB endothelium by altering TJ protein and myosin light chain (MLC) expression and phosphorylation.^60,61^ MLC activation directly influences endothelial permeability via actin modulation and indirectly facilitates paracellular passage via MLC kinase-mediated occludin phosphorylation. Aberrant AD mitochondria also release damage-associated molecular patterns (DAMPs) in cerebral ECs, which exacerbates vascular inflammation and BBB hyperpermeability.^62^

ALS cases and experimental models display mitochondrial pathology, and dysfunctional mitochondria impair BCNSB integrity in several disease contexts. These pieces of evidence strongly implicate mitochondria as mediators of BCNSB dysfunction in ALS. To better understand these mechanistic links, the following rescue experiments could be explored. Given that PGC1α overexpression reverses bioenergetic deficits seen in an iPSC model of ALS, it would be important to understand whether restoring mitochondrial function *in vivo* also results in improved BCNSB integrity.^52^ Similarly, since inhibiting the Drp1/Fis1 interaction using P110 improves mitochondrial function *in vitro*, this approach may also have effects on BCNSB health in animal models of ALS.^58^ The effects of restoring mitochondrial function at different stages of ALS would provide significant insight into the contribution of barrier pathology to motor neuron disease progression.

### Aberrant astrocytes

Similar to mitochondria, astrocytes are both altered in ALS and play important roles in maintaining BCNSB integrity. In ALS, astrocytes surround degenerating upper and lower motor neurons and show raised expression of inflammatory markers, including cyclooxygenase-2 (COX-2) and inducible nitric oxide synthase (iNOS).^63–65^ SOD1 rodents have astrocytic SOD1 protein inclusions and Cre-loxP systems suggest that selective ablation of the mutant *SOD1* transgene in astrocytes significantly slows disease progression and prolongs survival.^66,67^ In iPSC cultures, mutant SOD1 astrocytes release toxins and reduce motor neuron survival.^68^ Transplantation of healthy astrocyte precursor cells in SOD1 rats attenuates motor neuron loss and extends survival.^69^

In addition to their active role in ALS disease progression, astrocytes form the cellular connection between the BCNSB and neurons. As discussed previously, neurons, glia and microvessels are organised into neurogliovascular units, which regulate cerebral blood flow and BCNSB function at the capillary level.^9^ Astrocytes can alter the BCNSB phenotype in three broad ways, categorised by Abbott et al. into “physical”, “transport” and “metabolic” mechanisms.^70^ The physical barrier is determined by the permeability of TJs, which can be modulated by astrocytes via secretion of glial-derived neurotrophic factor (GDNF) and fibroblast growth factor (FGF).^71,72^ The expression of TJ proteins and mRNA is dysregulated in ALS. Endothelial cells express transporters, such as GLUT1 and Pgp, which allow specific substances to cross the barrier transcellularly and astrocytes support their expression and localisation.^70^ Several of these EC transporters are altered in ALS patients and models.^73^ Finally, specialised enzymatic pathways, including those involving cytochrome P450s, facilitate metabolic control of barrier function and are expressed in many BCNSB cell types.^74,75^ These pathways are disrupted in ALS, which has important implications for drug distribution in the CNS. Astrocytes thereby adopt several important functions in BCNSB homeostasis, which are altered in the state of chronic astrocyte reactivity seen in ALS.

In disease, reactive astrocytes both protect and disrupt BCNSB integrity.^76^ Astroglial scars help block the entry of pro-inflammatory molecules into the brain parenchyma and depleting astrocytes in inflammatory states worsens CNS pathology.^77^ Interestingly, astrocytes exposed to pro-inflammatory cytokines downregulate TJ protein expression at the BCNSB but promote the expression of claudin-1, claudin-4, and junction adhesion molecule-A (JAM-A). These proteins form tight junction-like structures at astrocyte end-feet in the glia limitans and restrict perivascular leukocyte infiltration.

On the other hand, microglial release of IL-1β, a pro-inflammatory cytokine, suppresses sonic hedgehog (SHH) production by astrocytes.^78^ This reduces their protective role at the BCNSB by downregulating TJ protein expression in ECs, which causes barrier leakage. IL-1β also promotes astrocytic release of vascular endothelial growth factor (VEGF) and pro-inflammatory chemokines, including CXCL2, CCL2 and CCL20, which exacerbate BCNSB dysfunction. Further, LPS challenges and reactive oxygen species induce astrocyte reactivity, suggesting astrocytic pathology may also contribute to the BCNSB abnormalities seen in previously discussed experiments focusing on mitochondria.^79,80^

Astrocytes express receptors for many neurotransmitters and modulators of BCNSB function, including those which increase BCNSB permeability, such as glutamate, and those which cause barrier tightening, like noradrenaline.^81^ One mechanism by which glutamate increases BCNSB permeability is by binding to N-methyl-D-aspartate (NMDA) receptors on ECs, which releases nitric oxide (NO).^82^ Astrocytes respond to NO by releasing astrocytic glutamate, which may cause further excitotoxicity and neuroinflammation.^83^ High-affinity glutamate transporters on astrocytes, GLT1 and GLAST, help protect neurons from excitotoxicity, but this function is likely overwhelmed in ALS.^84^ Some ALS patients have raised levels of glutamate in the CSF and glutamate-induced excitotoxicity is one mechanism which is believed to underpin motor neuron death.^85^ Riluzole, one of the few approved drugs for ALS, appears to have anti-excitotoxic effects by binding to voltage-gated sodium channels and limiting the axonal release of glutamate.^86^

In ALS, dysfunctional astrocytes lose the ability to promote neuronal survival and synaptogenesis, as well as their capacity to modulate the BCNSB phenotype and maintain barrier integrity. In particular, the signalling between astrocytes and ECs appears to be dysregulated in ALS. This pathology contributes to the early-stage BCNSB changes seen in ALS patients and animal models. Since transplantation of healthy astrocyte precursors appears to have protective effects on motor neuron survival in models of ALS, it would be of significant interest to determine whether BCNSB function is also improved.^69^ This would provide a more direct link between the astrocytic changes seen in ALS and BCNSB dysfunction. Similar experiments could assess BCNSB function following selective ablation of ALS-causing transgenes in astrocytes using Cre-loxP systems. These approaches could also be used to study changes in signalling, particularly via NO and glutamate, between astrocytes and ECs after astrocyte transplantation or transgene ablation.

### Neuroinflammation

Several aforementioned pathways converge to cause neuroinflammation in ALS, which exacerbates BCNSB damage. Neuroinflammation has widely been studied in ALS and is generally considered to be both a cause and a consequence of neurodegeneration. Initiation of ALS pathology in the motor cortex is characterised by early cerebral microglial and astrocytic reactivity, increased levels of pro-inflammatory cytokines and subsequent immune cell infiltration.^13^ Barrier dysfunction worsens this pathology as ALS disease progresses.

Three inflammatory mechanisms which affect BCNSB function in ALS are TDP-43 pathology, MMP activation and systemic inflammation. TDP-43 is a multifunctional transcriptional regulator which aggregates in the glial and neuronal cytoplasm in ALS and is implicated in key immune and neuroinflammatory pathways.^87^ TDP-43 deposits are also found in the basal lamina of small blood vessels in the spinal cord and frontal cortex of some ALS patients.^88^ These inclusions are not seen in ECs or pericytes. Overexpressing TDP-43 in cultured astrocytes incites secretion of IL-1β, IL-6 and TNF-α, which are elevated in the blood of ALS patients, and causes microgliosis via the pro-inflammatory NF-κB pathway.^89,90^ This study also found that expression of protein tyrosine phosphatase 1B (PTP1B), an inflammatory regulator, increases following TDP-43 accumulation. Notably, PTP1B inhibition reduces neuronal death and mitochondrial pathology induced by TDP-43-overexpressing astrocytes. The upregulation of cytokines which results from TDP-43 overexpression also recruits peripheral immune cells across the barrier. In TDP-43-overexpressing mice, marked infiltration of IgG and CD3+ and CD4+ T lymphocytes is associated with EC and pericyte reactivity.^91^ Extensive monocyte infiltration in the motor cortex is also evident in TDP-43-positive post-mortem tissue from ALS patients.^92^ Therefore, TDP-43 exerts detrimental effects on barrier permeability and neuroviability by disrupting BCNSB function.

The neuroinflammatory effects of TDP-43 are exacerbated by the release of MMPs. MMPs, produced by glial cells, are physiologically active at low levels to remodel and maintain the integrity of the basal lamina and regulate cytokines and chemokines.^93^ MMP activity is closely regulated to prevent excessive degradation of the basal lamina and cytokine-mediated immune cell recruitment into the CNS. When these regulatory mechanisms fail, such as in inflammation associated with TDP-43 aggregation and oxidative stress, serum levels of MMP-2 and MMP-9 rise. This is seen in both ALS patients and SOD1 models, where MMP dysregulation is linked to endothelial mitochondrial dysfunction, reduced capillary diameter and progressive loss of perivascular components, including occludin and collagen IV.^45^ These factors combine to increase BSCB permeability. While this study could not directly implicate MMPs in barrier component loss, BBB studies have demonstrated that TJ protein and basement membrane degradation is MMP-mediated.^94,95^ More concretely, MMP-9 knockdown protects motor neurons in both TDP-43 and SOD1 overexpressing mice.^96,97^ Similarly, in experimental autoimmune encephalomyelitis (EAE), a mouse model of CNS immune cell infiltration, astrocytes express MMP-2 and MMP-9, which enhances T-cell chemotaxis across the barrier and into the brain parenchyma.^98^ In ALS, astrocytic regulation of MMPs and basement membrane degradation may be dysfunctional due to the astrocyte reactivity seen in patients and disease models.

ALS patients also present with varying degrees of systemic inflammation, characterised by elevated levels of pro-inflammatory cytokines, including TNF-α, VEGF, IL-6, and IL-8, in peripheral blood.^99,100^ Wide-range C-reactive protein (wrCRP) and erythrocyte sedimentation rate (ESR) are also raised in ALS and correlate with disability, as measured using the ALS Functional Rating Scale-Revised (ALSFRS-R).^101^ This suggests that systemic inflammation is associated with worse prognosis in ALS. CRP activates microglia, increases BBB permeability and affects the complement system. Blood samples from ALS patients show further alterations in immune cell type and number, with increased lymphocyte and monocyte populations and a raised neutrophil:lymphocyte ratio.^102^ Persistent systemic inflammation remodels the BCNSB by altering receptor and carrier signalling and increasing cellular traffic along the barrier.^103^ One hypothesis underlying the link between systemic inflammation and BCNSB dysfunction is that primed microglia in NDDs are more sensitive to systemic inflammation.^104^ This theory is supported by the associations between systemic inflammation, BCNSB disruption and disease progression in AD and MS, which may also be true for ALS.

*In vivo* LPS challenges are used to model systemic inflammation. In some, but not all, studies, LPS disrupts the BBB by damaging ECs, modifying tight junctions, and causing structural and functional changes in astrocytes.^105^ LPS also exacerbates TDP-43 aggregation in astrocytes and microglia and induces mitochondrial dysfunction.^106^ This promotes neuroinflammation and motor neuron death in mouse spinal cords.

**Figure 3.**
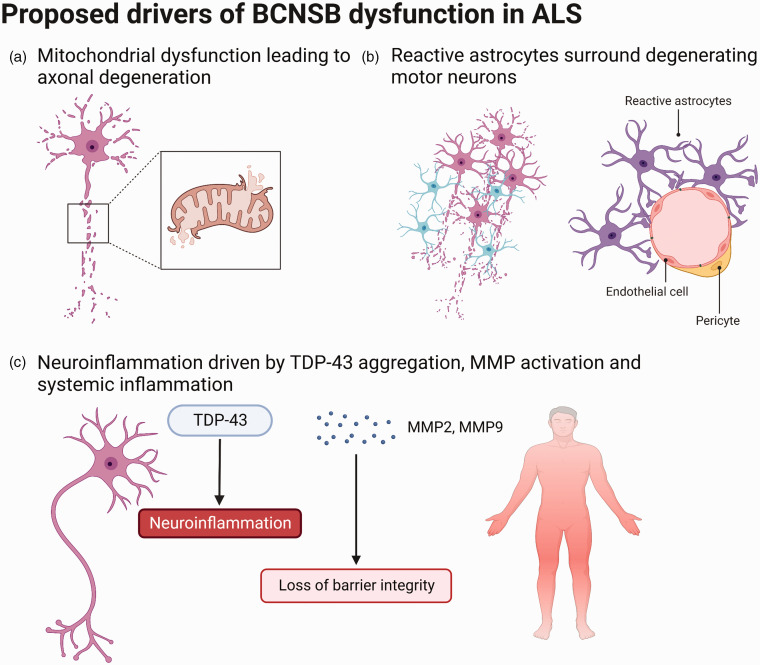
Proposed mechanisms driving observed blood-CNS barrier dysfunction in ALS, including (a) mitochondrial pathology prior to the onset of motor neuron degeneration, (b) astrocytes surrounding degenerating motor neurons and losing their functions at the BCNSB, and (c) neuroinflammation, in particular driven by TDP-43 aggregation, matrix metalloproteinase (MMP) activation and systemic inflammation. Adapted from “Endothelial Junctions in the Blood Brain Barrier” and “Histopathological features of Parkinson’s Disease and Alzheimer’s Disease”, by BioRender.com (2022). Retrieved from https://app.biorender.com/biorender-templates.

Together, this evidence suggests that neurological and systemic inflammation play important roles in ALS disease initiation and progression. As with mitochondrial dysfunction and astrocytic pathology, these inflammatory mechanisms provide a link between drivers of ALS disease and BCNSB dysfunction. The following approaches could be used to better understand how inflammatory cues cause BCNSB impairment in ALS. Since inhibiting PTP1B reduces neuronal death induced by TDP-43-overexpressing astrocytes, the effects of this intervention on BCNSB integrity and immune cell infiltration warrant further study.^89^ Additionally, MMP knockdown studies in ALS models will improve our understanding of the contribution of basement membrane degradation to the observed BCNSB abnormalities (Figure 3).

## Diagnostics and therapeutics

The BCNSB, due to its role in tightly regulating the movement of substances into and out of the CNS, is generally seen as a therapeutic challenge. However, the identification of BCNSB dysfunction in the early stages of many neurodegenerative diseases has generated interest in its diagnostic and therapeutic potential.

The typical time from ALS symptom onset to diagnosis is 10-16 months, which can involve misdiagnoses and unnecessary procedures.^24^ It is therefore hoped that biomarkers of disease severity and progression will shorten the delay to diagnosis and improve monitoring of treatment response in clinical trials. Two CSF neurofilaments, phosphorylated neurofilament heavy chain (pNfH) and neurofilament light chain (NfL), are released into the blood and CSF following axonal degeneration.^107^ PNfH and NfL are validated diagnostic biomarkers for ALS but more direct measures of BCNSB dysfunction could facilitate diagnosis prior to the onset of neuronal damage. A better understanding of the timing and mechanisms of BCNSB dysfunction in ALS would enable the development of blood and CSF biomarkers which exploit these abnormalities.

From a therapeutic perspective, direct manipulation of the BCNSB has demonstrated clinical potential for other neurological diseases, in particular epilepsy. For example, deep brain stimulation (DBS) of the anterior thalamic nuclei in rats, which is known to reduce seizures, also attenuates BBB disruption.^108^ Similarly, drugs which raise the expression of TJ proteins, such as ZO-1 and claudin-5, have been shown to restore BBB integrity in rodent models of epilepsy and AD.^109,110^ Inhibition of MMP-2 and MMP-9, both associated with BCNSB disruption in ALS, reduces seizure severity and spontaneous seizure frequency in two rat models of temporal lobe epilepsy.^111^

In addition, targeting mitochondria, astrocytes and inflammation in ALS may improve BCNSB integrity and thereby the disease course. Pre-clinical studies suggest that mitochondria, and in particular anti-oxidative stress pathways, are a promising therapeutic avenue in ALS.^52^ Although these mechanisms are likely systemic, the role of mitochondria at the BCNSB indicates this is an important component. Astrocytes are also a well-studied target in ALS. As discussed previously, loss of mutant SOD1 in astrocytes slows disease progression in ALS mice.^67^ In contrast, overexpression of the transcription factor Nrf2 in astrocytes has neuroprotective effects in culture and in ALS mouse models, which are mediated by astrocytic secretion of the antioxidant glutathione.^112^ Astrocytes are also implicated in the neurotoxic inflammation seen in ALS and upregulate transforming growth factor-β1 (TGF-β1), which inhibits the neuroprotective inflammatory response of microglia and T cells.^113^ Survival is extended in SOD1 mice when TGF-β signalling is inhibited. Further, activated protein C (APC), a serine protease with anti-inflammatory properties, corrects barrier integrity and decreases markers of toxicity in the CNS of SOD1 mice. In particular, APC administration at an early disease stage delays the onset of motor neuron degeneration and has been shown to downregulate mutant *SOD1* transcription in ALS neurons and microglia.^26,114^ APC also rescues motor neuron pathology, including impaired autophagosome formation and TDP-43 mislocalisation, in an iPSC model of ALS.^115^ This suggests that APC has protective effects on both BCNSB integrity and directly on neurons via its transmembrane signalling mechanism.

Finally, recent advances in the use of *in vitro* models of the neurogliovascular system may improve the screening of diagnostic and therapeutic targets. Microfluidic and microphysical platforms are commonly used in BCNSB modelling and produce functional blood vessels with TJ and matrix protein expression.^116,117^ 3-dimensional BBB organoids can be developed by co-culturing ECs, pericytes and astrocytes, which significantly improves upon models which culture ECs in isolation. In addition, BBB models which use human iPSC-derived brain ECs appear to recreate low paracellular permeability more accurately than primary brain ECs. IPSC models also have the advantage of deriving multiple BCNSB cell types from a single pool of cells.^118^ Together, these approaches will facilitate more efficient drug screening and development by using high-throughput techniques which consider the BCNSB phenotype in ALS. However, while tissue engineering methods are beginning to be used to investigate the BCNSB in neurodegenerative disease, there are few studies which specifically focus on ALS. So far, an iPSC model consisting of patient-derived astrocytes co-cultured with ECs has been developed.^119^ This model showed an upregulation of the efflux pump P-glycoprotein in ECs, which was driven by mutant SOD1 astrocytes, and this has been replicated in SOD1 rats.^120^

## Conclusions

Although the presence of BCNSB dysfunction in ALS has long been known, a better understanding of the disease-causing mechanisms which induce this pathology is required. In this review, we discuss how the mitochondrial pathology, aberrant astrocytes and neuroinflammation identified in ALS may help explain BCNSB changes seen in patients and experimental models. The basis of these hypotheses is that BCNSB abnormalities are a pre-symptomatic feature of ALS, which suggests a close biological association with the condition’s causes. Experiments which restore mitochondrial, astrocytic and neuroinflammatory health in models of ALS are required to further support the links between these pathologies and BCNSB dysfunction. BCNSB impairment in ALS also holds significant diagnostic and therapeutic relevance and advancements in the use of *in vitro* models will enable the neurogliovascular aspects of ALS to be studied further.
